# Modeling recreational visitation at Bureau of Land Management sites

**DOI:** 10.1038/s41598-026-43154-y

**Published:** 2026-06-17

**Authors:** Dieta Hanson, Spencer A. Wood, Sarah Rappaport, Emily J. Wilkins, Rudy M. Schuster

**Affiliations:** 1 Outdoor Recreation and Data Lab, University of Washington, Washington, USA; 2 U.S. Geological Survey, Fort Collins Science Center, Fort Collins, Colorado USA

**Keywords:** Outdoor recreation, Public lands, Visitation, Mobile device location data, Social media, Environmental sciences, Mathematics and computing

## Abstract

Estimates of recreational visitation are essential for public land management. Visitation is typically estimated using devices such as automated counters that require logistics and effort, particularly at remote locations or those with several access points. In this study, we investigate the utility of alternative data sources and statistical models for estimating visitation to public lands in the United States, through an analysis of data from 70 Bureau of Land Management sites. We compile 1328 site-months of visitor count data collected on-site, which are used to train and evaluate three random forest models incorporating combinations of 15 site-level characteristics and three sources of digital mobility data—mobile device locations, geolocated social media, and community science observations. Models including site characteristics perform better than models relying on mobility data alone. Cross-validation using held-out sites reveal varying prediction accuracy, suggesting that model generalizability depends on the inclusion of characteristically similar sites in the training data. These results underscore the limitations of relying solely on mobility data for visitation estimation and highlight the benefits of combining diverse data sources. Our approach provides a scalable, data-driven framework for estimating visitation where traditional monitoring is challenging or infeasible, supporting broader applications in recreation management.

**Supplementary Information:**

The online version contains supplementary material available at 10.1038/s41598-026-43154-y.

## Introduction

Monitoring recreational visitation on public lands is important for management and resource planning decisions made by the agencies that administer them, as well as for estimating the economic benefits of recreation^1–3^. Collection of accurate visitation data may also be legally required in some jurisdictions. In the United States, for example, the Expanding Public Lands Outdoor Recreation Experiences (EXPLORE) Act requires accurate annual visitation estimates for each unit of Federal recreation lands and waters^3^. Visitation, generally defined as the number of unique visitors during a specific time period, is typically estimated using on-site methods such as direct observation, automated counters, and administrative data generated by permit systems or fees^3^. However, these methods can be expensive and time-consuming as they require staff to physically visit sites to gather data, calibrate counts, develop multipliers (for example, converting vehicle counts to people counts), and monitor counting equipment for power, damage, and theft. This makes it challenging to implement these methods for estimating visitation at sites that are remote, difficult to access, or have multiple entry points that need to be monitored for visitors entering and exiting at different locations.

The United States contains many different types of parks, protected areas, and public lands managed by many agencies and organizations. The U.S. Bureau of Land Management (BLM) manages the largest area of public land, with 244 million acres (about one million km^2^)^4^. BLM lands support a variety of uses and receive over 80 million recreational visits per year^5^. These lands provide a diverse array of recreational opportunities, from hiking to off-highway vehicle driving, to visiting petroglyphs and fossil beds. There are also many dispersed-use sites within BLM lands, which are often large and remote areas with porous boundaries and without developed recreation facilities, where users recreate in a more self-directed manner than at a site with designated campgrounds and infrastructure. Given the extent of lands the BLM manages, many recreational opportunities are in remote areas where it is particularly challenging and time-consuming to collect on-site data. Despite its value to the public for outdoor recreation^5^, there has been little research studying recreation on BLM lands at a large scale. For example, of the four federal land management agencies that make up the National Wilderness Preservation System, the BLM administers 32.4% of all wilderness areas but is the focus of only 1.5% of research on visitor use^6^.

Research on methods for estimating visitation where on-site data collection is unfeasible has investigated the use of remotely-collected datasets, including geolocated social media, community science observations, and mobile device locations, collectively known as digital mobility data^7–9^. Geolocated social media generally includes content such as images and trip reports that indicates the presence of users in a specific location and time, and has been a commonly researched digital mobility data source for estimating recreational visitation^10–12^. Mobile device location data, which are geographical coordinates of mobile devices such as mobile phones, tablets, and smart watches, are most commonly provided by applications installed on the devices^3^. While mobile device location data are increasingly used as a proxy measure of visitation, few studies have examined the accuracy of this method or best modeling approaches for utilizing these data to estimate visitation to public lands at a large scale^3^.

Studies that have evaluated the usefulness of mobile device location data for measuring visitation observe positive correlations between the number of mobile devices and counts of people collected on-site in the same locations (e.g.^13–16^). These encouraging results, along with the perceived ease of obtaining visitation data without the need for field staff or specialized equipment, has made mobile device location data a particularly attractive option to recreation managers who need to generate visitation estimates on their lands with limited staff and resources. Mobile device location data also have generally higher sampling rates than other types of digital mobility data^9,17^, which makes it appealing for sites with low visitation rates. Nonetheless, a number of studies have identified issues and concerns, including temporal or spatial instability in the measured relationship between mobile device counts and actual visitation^9,18–21^.

A growing number of studies looking beyond correlations between digital mobility data and on-site visitor counts are researching whether these data are useful inputs into statistical models estimating visitation at outdoor recreation sites. The limited number of studies so far have concluded that visitation models can explain a majority of variance in visitation when (1) data collected on-site is used to parameterize the effects of the explanatory variables, and (2) the model includes a “site variable” that captures otherwise unexplained site-to-site variability. One study, for example, using mobile device location data to train a model for predicting visitation at 38 parks in the U.S. National Park Service system, found high correlation between observed and predicted visitation counts (conditional R^2^ = 0.964) when including a random effect for individual parks in their linear regression model^19^. A similar study included a fixed site effect in a model that used three different mobile device location datasets, as well as several other digital mobility datasets, to predict visitation at eight refuges in the U.S. National Wildlife Refuge System^9^. The majority of this model’s strong performance (adjusted R^2^ = 0.92) was due to the site variable, which explained differences in the magnitudes of visitation among sites. Furthermore, a model with site identity as the only predictor performed almost as well, suggesting that site-specific characteristics are powerful predictors of visitation, and potentially more predictive than variability in any digital mobility data.

To evaluate how visitation models may perform in real-world applications, predictions must be tested on data from sites beyond those used to train the model (known as “cross-validation” or “out-of-sample testing”). This approach gives a reasonable estimate of how accurately models trained on data from sites with counter data or other ground-truth data can predict visitation at other “unseen” sites with differing characteristics. This simulates an application of a model for estimating visitation at a site where on-site data collection is not feasible, and it means a fixed site effect cannot be included as a predictor in the model. One study performing cross-validation used on-site counts of visitors collected at trails in Washington to parameterize a model that was then used to predict visitation to trails in New Mexico, finding its performance to be too poor for use in practice (R^2^ < 0.5)^11^. However, this model only included variables for date, daily total precipitation, and user-days derived from digital mobility data, and the authors found that other models that explicitly allow for site-specific relationships (by including a site effect) between digital mobility data and visitation perform much better. While the study used a site effect to achieve that performance boost, this required having on-site visitor counts from all sites in order to train the model; to avoid the requirement of on-site counts from every site, which precludes the use of the model at novel sites, studies need to determine whether there are site-level variables that have generalizable relationships with visitation that can be used in place of a site effect. A more recent study developed a model for predicting visitation at U.S. National Park Service, U.S. Forest Service, and U.S. Fish and Wildlife Service parks, forests, and refuges, using the relationship between on-site counts of visitors and multiple digital mobility data sources, as well as a small number of site variables such as temperature, nearby population, and geographic region^17^. When the authors withheld whole sites from the training data—to cross-validate the model—they found 79.7% mean absolute percentage error in predicted visits. Both of these cross-validation studies ^11,17^ created a framework for cross-validating models developed on one set of sites to other potentially very different sets of sites. Building on this previous work, our goal is to test if it is possible to improve the out-of-sample performance of visitation models that use mobile device location data by including site variables that capture the site-to-site variability in the relationship between on-site counts and user-days of digital mobility data, avoiding the need for a site variable and accompanying on-site counts from each site.

In this study, we research whether digital mobility data and different predictor variables in visitation models improve estimates of visitation at sites on BLM lands. Given the lack of representative literature describing recreation on BLM lands, and the research opportunity provided by the diversity of site types and geographical spread that is represented by those lands, we designed this study to answer four research questions:

I. Are digital mobility data correlated with visitation at BLM sites? How strong are these correlations, and how much variability is there in correlations between sites at a monthly scale?
II. Are there measurable characteristics of sites that can account for site-to-site variability in visitation?
III. Given the best predictive visitation model, how well can monthly visitation be estimated at unmeasured (out-of-sample) BLM sites?
IV. What are the marginal contributions of predictor variables derived from mobile device location data in the best-performing visitation model?

## Methods

### Study sites

We studied visitation at 70 BLM recreation sites in ten out of 12 BLM administrative “states” to provide wide geographical representation (Supplementary Table S1). Sites are managed by staff in an administrative and geographical unit called a field office, which are in turn managed by district and state offices. The sites span 19 field offices, resulting in one to three field offices per state office in the dataset. The term “sites” refers to individual recreational destinations such as campgrounds, trailheads, and water-access points. We focused on estimating visitation at a site level since BLM recreation staff currently collect and report visitation data at that level—a reflection of how understanding small-scale visitation patterns can be informative for management. Relying on existing collaborations with BLM staff, we identified possible field staff to contact who may have collected accurate visitation data at small temporal scales (i.e., data at a monthly scale or finer). With those staff, we discussed the existing visitation data, including how data were collected, locations where data were collected, frequency of data collection, and perceived accuracy of the data at various sites and times. Perceived accuracy was based on whether staff applied rigorous methods including calibrating automated counters, adjusting counts to account for multiple access points, and developing multipliers (for instance, to convert from vehicles to visitors). We then worked with field staff to determine which sites within each field office would be suitable for inclusion in the study. Our criteria for site inclusion were: (1) there was a minimum of 4 months of on-site count data available (2) there was reasonable certainty that most of the visitors to the site were included in the counts and double-counting was controlled for and (3) if counts were of vehicles, there was an estimated multiplication factor from vehicles to people.

The selected sites represented a wide variety of types and sizes, from individual hiking trails to large areas used for off-highway vehicles (OHVs) or back-country camping (Supplementary Table S1). There was also a range of usage levels; some remote dispersed-use sites had only a few visitors per month, whereas other sites had large weekend events that attracted tens of thousands of visitors.

We worked with the BLM contacts to define the boundaries of each site by using geographic information system (GIS) software (QGIS version 3.32.3-Lima) to draw a polygon around the area in which visitors were counted. We excluded any area that could contain visitors not captured by the on-site counters, such as roads that might be used by people driving through and not stopping to recreate.

### On-site data

For each site, we used on-site visitor counts collected by BLM staff or contractors, aggregated to monthly total visits, where a visit is defined as one person entering a site. These counts were collected by one of four methods: infrared trail counters, magnetic vehicle counters, pneumatic tube vehicle counters, and gate counts (only used at one location which has controlled entry through a gatehouse). Counts from automated counters were calibrated by field staff to ensure accuracy. Most sites had one point of entry and exit, whether for a person (e.g., through a trailhead) or for a vehicle (e.g., on an entry road). With the counter placed at this point, staff ensured that everyone entering the site was counted. Some sites had secondary entry points that were not monitored, but based on field observations, visitors traveling through these points were a small fraction of total visitors (less than 5%). There were also some sites with counters at multiple entry points, and at these sites BLM staff used equations based on observational data to combine the counts into a single daily visit estimate without double-counting visitors. Finally, at sites that used vehicle counters, BLM staff used observational data to generate conversion factors from vehicles to people.

We received daily, weekly, or monthly on-site visit counts from BLM field staff, depending on the field office, covering the period from August 2021 to December 2023. We aggregated all on-site data to the monthly scale. For the daily data, we discarded the data from a month if there were missing data for more than 50% of days; for months when less than 50% of days were missing, we used the daily average of the remaining days to interpolate missing days, without any special accounting for holidays. After filling in any missing data, we then aggregated the daily counts to monthly totals. For the weekly data, we first divided the weekly counts evenly over the 7 days within the week, then followed the procedure for the daily counts: filling in missing days with the daily average (if less than 50% of days in a month were missing), then aggregating to month.

### Digital mobility data

We collected three digital mobility datasets to use in models predicting site visitation: one from a social media platform (AllTrails, also called “trail reviews”), one from a community science platform (eBird, also called “bird sightings”), and one from a mobile device location data vendor (Reveal, also called “mobile devices”). The data we collected spanned the same time period as the on-site data described above. AllTrails provides a website and mobile application that allows users to share reviews of outdoor recreation sites such as trails. These publicly available reviews, which we downloaded from the AllTrails website, are associated with unique user identifiers (ID), timestamps, and latitudes and longitudes representing sites (e.g., the start of a trail). Using the longitude and latitude of each AllTrails site, we intersected the AllTrails site locations with our BLM site polygons, such that one BLM site could be associated with reviews from multiple AllTrails sites. eBird is an online platform for people to report sightings of bird species—similar to AllTrails, each report is tagged with a unique user ID, a timestamp and the latitude and longitude of the reported sighting. We downloaded the “eBird Basic Dataset” for the relevant time period, which contains all the geolocated checklists in the United States (where a checklist contains all of a user’s sightings for a specific time period and location), and is publicly available for non-commercial use from the eBird website^22^. AllTrails reviews and eBird entries are not created by users for the purpose of estimating visitation, but nonetheless may be useful predictors for modeling variability in visitation.

Finally, we purchased a mobile device location dataset (the “VISIT” product) from the company Reveal. This contained an anonymized device ID, timestamp, and latitude and longitude for mobile devices (mostly mobile/cellular phones) in the vendor’s dataset. While Reveal processes the data to remove duplicates and low-quality data, they do not otherwise manipulate the raw location data. The vendor does not share specific details about the origins of the data, but in general terms these data are generated by applications (“apps”) running on mobile devices that use location-based services. This is often done through a software development kit, which is embedded in the application and interfaces with device’s global positioning system hardware to determine the location of the device. Devices must obtain permission from the users to access and collect the location data. We intersected the site polygons with all three of these datasets to select the data occurring within the sites. For each dataset, we then counted the number of unique user/device IDs per day per site (user-days^23^) and finally summed these user-days over each month to calculate total monthly user-days per site per platform. Therefore, as with the on-site data, the mobility data represented a monthly aggregate of daily counts.

We refer to the three mobility datasets as “trail reviews”, “bird sightings”, and “mobile devices” in order to avoid any appearance of governmental sanction and endorsement of the data provider^24^. None of the digital mobility datasets are considered to be a measure of absolute visitation, but rather a subset of visitation that may be correlated with and predictive of absolute visitation. The magnitude of this correlation and the importance of mobility data in visitation models are two main research questions addressed by this study.

### Site characteristics

We developed a list of 15 characteristics of sites to include in a model used to predict monthly visitation. This list was created based on what prior studies have found to be significant predictors of visitation^17,20,25–28^, the availability of the variables, and our hypotheses about which aspects of a site might be correlated with visitation. The characteristics are: site type, Recreation Management Area type, area, distance to nearest urban center, distance to nearest primary road, temperature, precipitation, population within 50 miles, road length within the site, presence of parking areas, presence of toilet facilities, presence of tourist points of interest, presence of water features, site activities, and developed land. Each variable and its full description is listed in Supplementary Table S2.

### Analyses

#### Correlation analysis

To evaluate the utility of digital mobility data as predictors of site visitation, we calculated Pearson’s correlation coefficients describing the relationship between data from each platform (bird sightings, trail reviews, and mobile devices) and on-site counts, both overall and by site. All values were log-transformed before calculating correlation coefficients in order to achieve bivariate normality in the data.

#### Modeling

We developed three random forest models to estimate visitation at BLM sites and to test the ability of our measured site characteristics to capture differences in visitation across sites (Table 1) Random forest models are ensembles of decision trees widely used in quantitative applications, that can effectively describe linear and nonlinear effects of a large number of predictors on a response variable^29,30^. Monthly on-site counts were the dependent variable in each model. The independent variables in Model 1 (“Baseline”) were the three digital mobility datasets and a categorical variable for site ID. This model served as a baseline to compare to Model 2 (“Site characteristics”), where we replaced site ID with the site characteristics as well as categorical variables for state office, month (January through December), and year. We hypothesized that if our site characteristics were effectively capturing the variability in visitation explained by site, the model performance of Model 2 would be the same or better than that of Model 1. Following this, to evaluate if our site characteristics were not fully explaining the variability in visitation due to site, Model 3 (“Site characteristics + site ID”) reintroduced site ID to the model, while keeping all the site characteristics. Here we hypothesized that adding site ID back in Model 3 would not improve model performance relative to Model 2, if our site characteristics were capturing all variability that is due to site.

**Table 1:** Model names and forms for the three random forest models used to predict monthly visitation at 70 different sites on Bureau of Land Management lands. For information on all site characteristic variables, refer to Supplementary Table S2.

Model	Model form
(1) Baseline	On-site_monthly_visits ~ monthly_userdays_mobile_devices_ + monthly_userdays_trail_reviews_ + monthly_userdays_bird_sightings_ + site_id
(2) Site characteristics	On-site_monthly_visits ~ monthly_userdays_mobile_devices_ + monthly_userdays_trail_reviews_ + monthly_userdays_bird_sightings_ + state_office + month + year + site_type + rma_type + area + urban_distance + road_distance + mean_temperature + mean_precipitation + population_50_miles + road_length + parking + toilet + tourism + water + activity_profile + developed_land
(3) Site characteristics + site ID	On-site_monthly_visits ~ monthly_userdays_mobile_devices_ + monthly_userdays_trail_reviews_ + monthly_userdays_bird_sightings_ + state_office + month + year + site_type + rma_type + area + urban_distance + road_distance + mean_temperature + mean_precipitation + population_50_miles + road_length + parking + toilet + tourism + water + activity_profile + developed_land + site_id

For each model, we determined the optimal model parameters *mtry* (the number of variables to possibly split at in each node in a decision tree) and *min.node.size* (the minimal node size to split at in each decision tree) using the ranger function implemented in the caret package for R^31–33^. This was done by testing every possible combination of potential parameter values; for each model, for each combination of model parameters, we ran a five-fold cross-validation routine with 2000 trees per fold^32^. This standard approach takes 20% of the data (known as a fold) and withholds it while using the remaining 80% to train a model on a given set of model parameters. Once the model is trained, it is tested on the withheld 20% to evaluate the chosen parameters. This process is then repeated four more times with the same model parameters, each time with a new 20% of the original dataset, before repeating the entire process (testing on 20% of the dataset five separate times) for each set of parameters^34^. We then used the best-performing set of model parameters for each model, determined by the lowest average root-mean-square error (RMSE) across the five folds per parameter set, to generate predictions for the entire dataset and used the RMSE as a measure of in-sample model performance.

Next, we selected the best of the three models (defined by the lowest RMSE) and performed an out-of-sample cross-validation to evaluate the ability of the model to predict visitation at new sites (i.e., sites not included in the training data). To do this, we randomly selected 20% of sites to exclude from the training data and trained a random forest model on the remaining 80% of sites, using the hyperparameters identified in the first cross-validation routine. We then repeated this process four more times, each time selecting another random 20% of sites to exclude (without replacement). Our final measure of model performance on new sites was the average RMSE across these five folds.

#### Importance of mobile device location data

To assess the relative importance of the mobile device location dataset for predicting visitation, compared to the other digital mobility datasets (our fourth research question), we used the “permutation” method of calculating variable importance in the ranger package for R^31^. This method is commonly used to assess variable importance in random forest models and works by randomly permuting the values of a given variable and comparing the model performance with the permuted values to one with the original values. A larger decrease in model performance due to permuted values indicates a greater importance. We calculated variable importances for the best-performing model, chosen as described in the “Modeling” section above.

## Results

### On-site and mobility data

In our final dataset, we had complete data for 1328 site-month combinations. The time period covered by the on-site data varied by site, from between four and 28 months. Across all sites the on-site data covered the timespan between August 2021 and December 2023. Digital mobility data from all three data sources were available for each site for the entire timespan (August 2021 to December 2023). Visitation according to monthly on-site counts varied widely from site to site, as did the number of monthly user-days from the digital mobility data, which also showed wide variability among platforms (Table 2).

**Table 2:** Minimum, maximum, median, mean, and standard deviation values of monthly on-site counts and digital mobility data user-days at the site level across the 70 sites in our sample.

	Minimum	Maximum	Median	Mean	Standard Deviation
On-site counts	0	73,457	884	3500	7130
Mobile device user-days	0	1858	11	88	220
Bird sighting user-days	0	71	0	0.88	3.87
Trail review user-days	0	340	1	13	33

### Correlation analysis

**Fig. 1:**
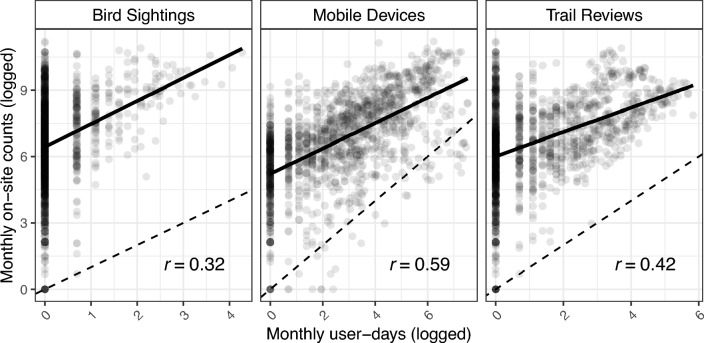
Overall correlation. Correlations between logged monthly on-site counts and logged monthly user-days across all 70 sites, by platform. User-days data from bird sightings (left panel), mobile devices (center panel), and trail reviews (right panel). Solid line shows the linear best-fit, dashed line indicates 1:1 ratio and the Pearson’s correlation coefficient is indicated in bottom right corners.

Our correlation analysis of on-site and digital mobility data found that correlations were moderate and varied among platforms and sites, each with a sample size of 1328 site-months. The highest overall correlation was with mobile devices which had a Pearson’s coefficient of r = 0.59, while trail reviews and bird sightings were less correlated, with coefficients of r = 0.42 and r = 0.32, respectively (Fig. 1).

**Fig. 2:**
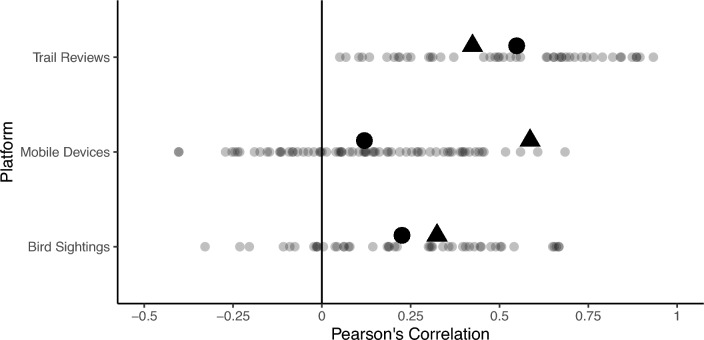
Site-level correlations. Site-level Pearson’s correlation coefficients between logged monthly on-site counts and logged monthly user-days, by each digital mobility platform. For each dataset on the y-axis, small points represent the individual site correlations (for 70 different sites), large points represent the mean of the individual site correlations, and triangles represent the overall correlations (not considering site).

At a site level, correlations showed different patterns, with trail reviews having the highest average site-level correlation, followed by bird sightings, and mobile devices with the lowest (Fig. 2). For each mobility data source, correlations varied widely from site to site, with all sources having correlations ranging from below zero to above r = 0.5 (maximums of r = 0.89, r = 0.74, and r = 0.67 for trail reviews, bird sightings, and mobile devices respectively) (Fig. 2).

### Modeling

**Fig. 3:**
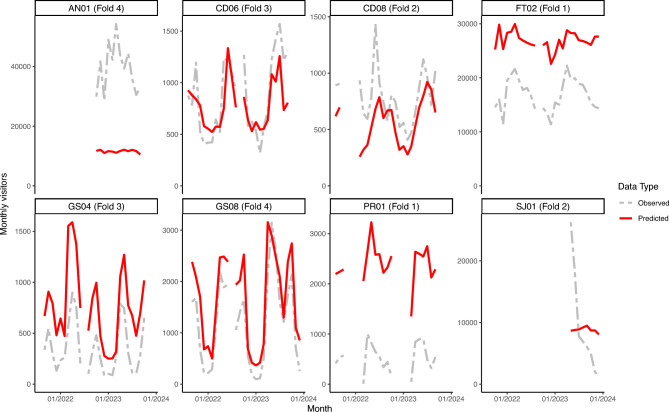
Selection of model predictions. Model 2 (which included site characteristics, but not site ID) predictions of monthly visitors for a selection of sites that were withheld from the training data. Dashed grey line shows observed values and solid red lines show model predictions. Note the scale of the y-axis differs among panels.

**Fig. 4:**
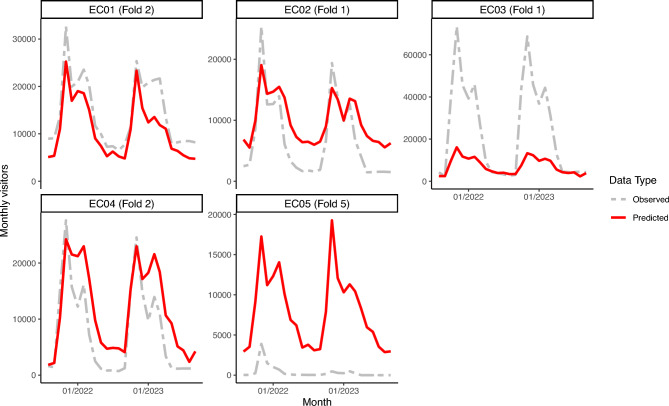
Model predictions for sites within El Centro Field Office. Model 2 (which included site characteristics, but not site ID) predictions of monthly visitors for all the studied sites in the El Centro Field Office. Each site was withheld from the training data. Dashed grey line shows observed values and solid red lines show model predictions. Note the scale of the y-axis differs among panels.

Using the optimal hyperparameters determined by the grid search, Model 1 had an in-sample RMSE of 2020 visitors, Model 2 had a lower RMSE of 931 visitors, and Model 3 had an RMSE of 940 visitors. This suggests that our site characteristics effectively captured the variation in visitation due to factors associated with the site. Since Model 2 (which included site characteristics, but not site ID) had the best in-sample performance, we used this model in the five-fold cross-validation routine with 20% of sites left out in each fold. The average RMSE of that cross-validation was 5152 visitors, but there was wide variance in RMSE across folds (8551, 5219, 1847, 6395, and 3748 visitors per fold: normalized by the standard deviation of the observed on-site visitor counts, these values are 0.801, 0.778, 0.856, 0.770, and 1.50). Some sites had relatively accurate predictions, whereas other sites showed large differences between predicted and actual values—mostly due to differences in scale (Supplementary Fig. S1). Figure 3 shows predictions for a selection of sites that come from field offices with a varying number of sites, showing the variability in prediction accuracy from site to site, and Fig. 4 shows the predictions for all the sites from a single field office (El Centro Field Office in California).

### Variable importance

**Fig. 5:**
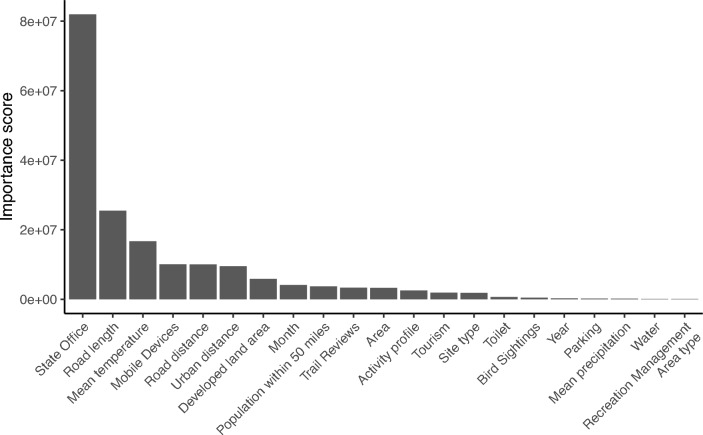
Variable importance. Importance scores from the variable importance analysis of all predictor variables included in Model 2, predicting monthly visitation to 70 recreation sites. For a description of variables, refer to Supplementary Table S2.

The variable importance analysis showed that of the different digital mobility sources, the mobile device data improved Model 2 performance the most, followed by the trail review and the bird sighting data, which had importance scores three- and 21-times smaller than the mobile device data, respectively (Fig. 5). Mobile device location data were also the fourth-most important feature overall, after state office, road length, and mean temperature. State office was a dominant predictor, with an importance score over three times larger than the next most important variable (road length).

## Discussion

### Model performance

Through a comparison our three alternative visitation models, we learned that certain site characteristics such as road infrastructure (as measured by total road length within the site) and average monthly temperature are predictably related with visitation, as is the state office (a geographical and administrative region very similar to U.S. states) in which the site is located. When these variables are included in predictive models along with predictors derived from digital mobility data, they explain substantial amounts of site-to-site variability in visitation (Model 2) and greatly improve in-sample model performance when compared to a model without those characteristics (Model 1). Further, re-introducing a site variable to a model with site characteristics (Model 3) does not increase in-sample model performance, suggesting that these site characteristics describe the majority of site-to-site variability in visitation. This result moves the research closer towards the goal of developing visitation models that can be applied at recreation sites with little or no on-site visitor count data, as long as on-site counts are available from other characteristically similar sites. Such models could be most beneficial for estimation in places where it is logistically challenging or expensive to collect on-site counts (e.g., remote areas that are hard for staff to regularly access). For example, if a field office has some sites where on-site counts are easy to collect and other characteristically similar sites where it is more difficult, one approach would be to build a model using the former sites and use it to estimate visitation to the latter sites.

### Variable importance

Our variable importance analysis of Model 2 provides insights into which variables are most important to accurately model visitation across all the study sites. The most important variable by far—with an importance score more than triple the next most important variable—is the state office in which the site occurs. This variable captures regional differences in visitation (including differences in recreation management among state offices) and acts as a proxy for regional interactive effects on visitation such as weather. For example, cold temperatures and high precipitation in Alaska could affect visitation differently than the same conditions in California. Additionally, since our data only represent one to three field offices per state office, the state office variable could be capturing some field office effect (especially as field offices within the same state office tended to be similar to one another in terms of geography and site types). It is possible that state office may be of lesser importance in future models trained on data from many more sites across the United States, representing many field offices per state office. After state office, the next most important model variable is the total length of roadways in the site, consistent with findings from other studies and locations that have observed that road length is associated with visitation^25^. We hypothesize this variable is acting as a proxy for either the amount of built infrastructure at the site or the ease of visitors being able to access areas within the site, although the mechanics of the relationship between visitation and roadway length is difficult to assess; there is no clear linear relationship between the two. The distance between the site and the nearest major road is also relatively important in the model. In this case there is a linear relationship between visitation and the variable, with sites at a greater distance from a major road having lower visitation, indicating this variable could be a proxy for accessibility or travel time to reach the site.

Compared with other variables, site type—which describes the general use of the site—is relatively unimportant for estimating visitation in the model. This could be due to the site type categories being too broad and not meaningfully related to visitation. For example, both sites AN01 in Alaska and FA03 in New Mexico are categorized as dispersed use sites, however the former site is urban-proximate and receives 39,000 visitors per month, while the latter site is relatively remote, receiving an average of only 1000 visitors per month. We also found that other site attributes such as tourist points, toilets, parking, and water features were less important predictors. While it is possible these variables have limited effects on visitation, it is also possible that the data are incomplete as they were derived from unofficial data available through OpenStreetMap. Because these data are dependent on volunteers to tag features, sites that do not receive attention from volunteers will have less data, a downside of relying on crowd-sourced data. Future models could likely remove these variables without much effect on model accuracy, which would also ease the burden of data collection.

One of our research objectives was to understand the marginal contribution of mobile device location data in predictive visitation models. Out of the three digital mobility datasets, the mobile device data had the highest importance score in Model 2, although it was still less important than state office, total road length within the site, and mean monthly temperature. The relatively low importance of mobile device data could be due to the large variability in correlation between on-the-ground counts and mobile device user-days across sites. It is possible that mobile device data may have more importance in a model including a site effect that would account for that variability. These findings, and the relatively poor performance of Model 1 with digital mobility data as the only predictors, suggest that visitation models should not depend solely on this type of mobility data. Rather, mobility data are useful as one component of a model that relies on many other data sources that describe factors known to affect visitation, as other recent modeling studies have found^3,9^. Certainly, because the number of mobile devices that appear at any given site represent a biased subset of visitors, and because this bias differs from site to site, it is not a reliable proxy for actual visitation.

### Estimation at out-of-sample sites

We find wide site-to-site variability in how well Model 2 can estimate visitation to out-of-sample sites—an analysis that mimics the task of applying a model that is trained on data from one set of locations to other places that lack any on-site visitation data. As shown in Fig. 3, Model 2 can predict visitation to some sites such as GS08, CD06, GS04, and CD08 with relatively high accuracy compared with others such as AN01, SJ01, FT02, and PR01. We propose three potential reasons for this variability. First, it could be due to the site-to-site variation we observe in the strength of the correlative relationship between digital mobility data and on-the-ground counts (Figs. 1 and 2). While the overall correlation between actual visitation and the three digital mobility datasets is positive, there is a wide range of correlational values at the site level, indicating that mobility data is more useful for predicting visitation at some sites relative to others (although other, non-linear relationships with visitation may be present and useful for predicting visitation). This is consistent with much previous research observing that mobility data are strongly correlated with visitation at some sites but not others^9,19,21^. The wide range in correlation could be due to many factors, such as sample bias related to how mobility data are typically collected via mobile applications. For instance, some applications might be more or less popular at different sites, leading to different detection rates. Differences in cellular service between sites may also impact data sharing by mobile applications that do not cache location data when there is no reception^3^. This finding indicates that mobile device locations and other digital mobility data would not be expected to give accurate visitation estimates on their own and are best used as one component among many in a visitation model.

A second potential explanation for the variable accuracy in out-of-sample visitation prediction is that sites used to train the model can be characteristically different from sites used to test its performance. We observe that sites with the most inaccurate predictions are also often the sites for which we lack other sites from the same field office. Given that sites we studied that occur within the same field office tend to be more similar to each other than to sites in different field offices, a model parameterized without any sites from a given field office may not capture the relationships between the predictors and visitation that are unique to that field office or region. Site AN01, for example, which is poorly predicted (Fig. 3), is the only site included in the study from the Anchorage Field Office, and the only site from the Alaska State Office. Without data to train the model on visitation patterns in that region, it may not capture relationships between visitation and combinations of variables that would only be found in Alaska—for example, high levels of visitation even in cold weather. Conversely, when sites from the same field office were included in the training data, predictions were generally better (see CD08, GS04, CD06, and GS08 in Fig. 3).

Finally, besides field office, variation in model accuracy among sites may be due to other relationships not included in the training data. For example, two of five sites within the El Centro Field Office were poorly predicted (Fig. 4). Those two sites had much higher (EC03) and much lower (EC05) average visitation than the other sites, which suggests that either the relationship between visitation and the model variables found at EC03 and EC05 were unique to those sites, and so were not included during model training, or that we did not have variables included in the model to accurately capture what was driving visitation levels at those sites.

## Study limitations and future work

Further investigation into the ways that site characteristics affect visitation could improve our understanding of where and when on-site data collection should be done to get the greatest and most cost-effective improvements in model performance. The evidence that visitation is better estimated at sites that are less unique within a region suggests that visitation models will perform best when parameterized using on-site data from similar or nearby sites. Future work could focus on quantifying the trade-off between the cost and time needed to collect on-site data for model training and model accuracy. This would be especially valuable to recreation managers with limited resources who need to decide what level of accuracy in visitation estimates they can accommodate.

As more visitation models are developed, the ability to compare them to previously published models is important for advancing the field and for providing practitioners who may want to implement them with the data necessary to make an informed decision. Due to differences in the scale of visitation, we cannot compare the RMSE values from our model to those reported for the few other studies that have done out-of-sample tests^11,17^, and the scale-independent metric of mean absolute percent error (MAPE) cannot be calculated due to zeros in our data. However, normalized RMSE (NRMSE) values are an option that would be easy to implement in future studies and provide a clearly defined, scale-independent measure of model error, which motivated their inclusion in the results of the present study.

This study evaluates whether on-site visitation is related to three variables derived from digital mobility data: the numbers of unique mobile devices, social media users who post trail reviews, and community scientists who post bird sightings. All three variables are a measurement of “user-days” of mobility data^23^, which is the most commonly studied method^3^. However, many alternative variables could be derived from these mobility data sources. Future research could test alternative variables based on, for example, total time spent at a site, or create variables that reflect different user groups based on characteristics of the users. These characteristics could be based on the spatial movement of the users or in the content of their social media posts.

Finally, all visitation estimates have some degree of inaccuracy and uncertainty. For these models, we only trained and tested models using on-site data with very high perceived accuracy. There is a question of how uncertainty in on-site estimates affects visitation model performance and if including less-certain on-site data would potentially be a detriment.

## Conclusions

Understanding how many visitors use an outdoor recreation area is important for making informed visitor-use management decisions. However, field staff experience challenges with monitoring and counting visitors, including technological difficulties (e.g., traffic counters being broken, vandalized) as well as the amount of staff time required to regularly check the functionality of counters and retrieve data. As a result, decision-makers within land management agencies are exploring new methods and alternative sources of data for estimating recreational visitation on public lands and waters that could be more efficient and cost-effective than traditional methods^3,8^.

We found that on its own, mobile device location data show such a wide range of correlation with actual monthly visitation that to use it directly as a measure of visitation would produce highly uncertain estimates. This is in line with many studies that have found that mobile device location data should not be used “off the shelf” to measure recreational visitation, but rather used in conjunction with other data either as a model input or for calibration and validation^3,8,9,19–21,35^. Additionally, staff may face difficulties determining what mobile device location data provider would best suit their needs, and adopting use requires learning technical skills that ultimately may not save staff time. However, given that on-site counting using established methods such as trail counters is not possible at all sites, this study asks whether and how mobile device location data could be a valuable tool for field managers generating visitation estimates at such sites. Our findings demonstrate that mobile device location data have the potential to improve visitation models that can be applied across a relatively wide array of recreation sites on public lands, but only when those models include other predictive variables such as temperature and site characteristics, and when models are calibrated with on-site data from other sites.

This study advances our understanding of how to estimate visitation at sites where field staff have not collected on-the-ground visitor counts. We explicitly evaluate the performance of a generalizable model through out-of-sample testing that mimics how a visitor estimation program could be operationalized by land managers. To implement a model like the one tested in this study, our findings suggest that managers could benefit from further research and development of a model at a national scale with geographic predictor variables to capture regional variability in visitation. Generally, our results indicate that models would perform better with more on-the-ground training data, as expected, likely because this expands the range over which the model parameterizes effects of predictor variables and therefore trains models that work across a variety of site types. With these results in mind, the development of visitation models holds promise as a source of data for managers who need to understand visitor use when making important resource-use decisions.

## Supplementary Information

Supplementary Information.

## Data Availability

All data and code used in this study is available on the Open Science Framework (OSF) platform at 10.17605/OSF.IO/3ACB9.
