# Supplementary material for: Modeling recreational visitation at Bureau of Land Management sites
Source: Sci Rep. 2026 Jun 17;16:18848. doi: 10.1038/s41598-026-43154-y (PMC13276051; doi:10.1038/s41598-026-43154-y)
Supplement: Supplementary file 1 — Supplementary Information. [file 41598_2026_43154_MOESM1_ESM.pdf]

Modeling recreational visitation at Bureau of Land Management sites

Dieta Hanson[1]*, Spencer A. Wood[1], Sarah Rappaport[2], Emily J. Wilkins[2], Rudy M. Schuster[2]

[1] Outdoor Recreation & Data Lab, University of Washington, Washington, USA

[2] U.S. Geological Survey, Fort Collins Science Center, Fort Collins, Colorado, USA

*Email address: dietah@uw.edu

Any use of trade, firm, or product names is for descriptive purposes only and does not imply endorsement by the U.S. Government.

Supplementary Information

Supplementary Table S1. Bureau of Land Management study sites and their respective state office, field office, and site type.

State Office	Field Office	Site Name	Site Type
Alaska	Anchorage Field Office	AN01	Dispersed use
Nevada	Basin and Range National Monument	BR01	Geological
		BR02	Archaeological
		BR03	Archaeological
	Red Rock Canyon/Sloan Canyon National Conservation Area	RR01	Trailhead
		RR02	Dispersed use
		RR03	Trailhead
		RR04	Trailhead
Montana-Dakotas	Butte Field Office	BU01	Day use
		BU02	Campground
		BU03	Campground
		BU04	Campground
		BU05	Campground
		BU06	Campground
		BU07	Campground
Wyoming	Cody Field Office	CD01	Water access
		CD02	Trailhead
		CD03	Trailhead
		CD04	Trailhead
		CD05	Day use
		CD06	Trailhead
		CD07	Water access
		CD08	Trailhead
		CD09	Campground

	Buffalo Field Office	BF01	Day use
Colorado	Uncompahgre Field Office	UN01	Trailhead
		UN02	Day use
	Royal Gorge Field Office	RG01	Trailhead
		RG02	Dispersed use
		RG03	Day use
		RG04	Trailhead
		RG05	OHV use
Utah	Moab Field Office	MB01	Trailhead
		MB02	Trailhead
	Price Field Office	PR01	Geological
	Grand Staircase-Escalante National Monument	GS01	Archaeological
		GS02	Geological
		GS03	Trailhead
		GS04	Trailhead
		GS05	Trailhead
		GS06	Trailhead
		GS07	Trailhead
		GS08	Trailhead
		GS09	Dispersed use
Washington-Oregon	San Juan Islands National Monument	SJ01	Trailhead
	Cascade-Siskiyou National Monument	CS01	Nordic ski
		CS02	Trailhead
		CS03	Trailhead
		CS04	Nordic ski
		CS05	Trailhead
		CS06	Trailhead
Arizona	Vermillion Cliffs National Monument	VC01	Dispersed use
		VC02	Geological

		VC03	Archaeological
		VC04	Geological
		VC05	Geological
California	Fort Ord National Monument	FT01	Trailhead
		FT02	Trailhead
	Santa Rosa and San Jacinto Mountains National Monument	SR01	Day use
	El Centro Field Office	EC01	OHV use
		EC02	OHV use
		EC03	OHV use
		EC04	OHV use
		EC05	OHV use
New Mexico	Farmington Field Office	FA01	Trailhead
		FA02	Trailhead
		FA03	Dispersed use
		FA04	Trailhead
		FA05	Dispersed use
	Las Cruces District Office	LC01	Archaeological

Supplementary Table S2. Site characteristics used in Model 2 and Model 3.

Name	Description
Site type	This variable described the way the site is generally used by visitors. We started with the site-type designation from the BLM's internal Recreation Management Information System (RMIS) and then modified these designations in two ways. First, for sites that were not in RMIS, we used information from the BLM field staff and from any published site information to categorize those sites into one of the existing types. Second, we recategorized some of the sites to ensure consistency across field offices or to consolidate similar site types. For example, one site was the only site designated as "historical" in our sample, so we recategorized it as "archaeological", since there were four other sites with this designation in our sample and they describe similar uses.
Recreation Management Area (RMA) type	Each site recorded in the BLM RMIS was given one of three RMA designations (extensive, special, or none), which are based on recreation demand and issues, site characteristics, and resource protection needs. For sites not in RMIS, we used the RMA designation of the nearest RMIS site within the same field office.
Area	The area of the site in square meters.
Urban distance	The distance in meters from the centroid of the site polygon to the nearest city with a population of at least 100,000 people.
Road distance	The distance in meters from the centroid of the site polygon to the nearest point of a primary road, as defined in the Topologically Integrated Geographic Encoding and Referencing shapefile published by the U.S. Census Bureau[1].
Mean temperature	The monthly average temperature in Celsius, calculated using the average across all 0.1 x 0.1 degree cells from the ERA5-Land monthly average 2 m temperature dataset occurring in the site polygon[2].
Mean precipitation	The monthly average precipitation in meters, calculated using the average across all 0.1 x 0.1 degree cells from the ERA5-Land monthly average precipitation dataset occurring in the site polygon[2].
Population within 50 miles	The population living within 50 miles (81 km) of the site (drawn as a 50-mile buffer from the site boundary). For the population data, we used the map of the 2020 Dasymetric Population for the Conterminous United States, Alaska, Hawaii, Puerto Rico, and the US Virgin Islands[3], which estimates population density for each 30 m by 30 m cell covering the United States.
Road length	The total distance of all roads, streets, and paths within the site. We measured this by querying OpenStreetMap[4] for all features with the key value of "highway" using the OSMnx Python package[5], then summing the lengths of all returned features.
Parking	Binary variable indicating if there was at least one parking area within the site boundaries. We queried OpenStreetMap[4] using the OSMnx Python package[5] for the key-value pair of "amenity:parking". If any value was returned, we assigned a value of "1" to the site or otherwise assigned "0".
Toilet	Binary variable indicating if there was at least one public toilet within the site boundaries. We queried OpenStreetMap[4] using the OSMnx Python package[5] for the key-value pair of

	"amenity:toilets". If any value was returned, we assigned a value of "1" to the site or otherwise assigned "0".
Tourism	Binary variable indicating if there was at least one feature tagged as being of interest to tourists within the site boundaries. These features fell into a wide range of categories and included informational signs, viewpoints, camping sites, or attractions such as caves, petroglyphs, rock formations, etc. We queried OpenStreetMap[4] using the OSMnx Python package[5] for any feature with the key value of "tourism". If any value was returned, we assigned a value of "1" to the site or otherwise assigned "0".
Water	Binary variable indicating if there was at least one natural water feature (such as a river or lake) within the site boundaries. We queried OpenStreetMap[4] using the OSMnx Python package[5] for any feature with the key value of "water". If any value was returned, we assigned a value of "1" to the site or otherwise assigned "0".
Activity profile	To characterize the types and relative popularity of different activities that visitors to sites participate in, we used activity participation data estimated by the recreation staff at each site for RMIS. These data covered the period from October 2022 to September 2023 and included the estimated number of annual participants in each of 49 different activity types. We first created a new variable "activity_group" by consolidating similar activity types together into 23 groups (e.g., the types "Viewing - Cultural Sites", "Viewing - Other", "Viewing - Scenery/Landscapes", "Viewing - Wildflowers", and "Viewing - Wildlife" were all combined into one group called "Viewing"). We then calculated the proportion of all activity participants occurring in each activity group, by site. Finally, to reduce the dimensionality of these variables, we performed a principal components analysis on the group proportions and extracted the first principal component, which explained 27% of the total variance (exploratory model building indicated that including additional components did not improve model performance). Because we used proportions of each activity instead of raw values, much of the effect of scale should be removed from this principal component, and it should represent the general types of activities that are present at a site and their relative popularity.
Developed land area	The percentage of the site area that is classified as "Developed" according to the National Land Cover Database published by the U.S. Geological Survey. We used the 2019 product for the sites within the conterminous U.S.[6] and the 2016 product for the site in Alaska[7]. For each product, we intersected the raster file of land use cover with the site polygons in QGIS[8], then used the "zonal histogram" tool to extract the number of cells classified as "Developed" (one of "Developed, High Intensity", "Developed, Medium Intensity", "Developed, Low Intensity", or "Developed, Open Space") in each polygon. We then divided this number by the total number of cells in each polygon to get a percentage.

[1] U.S. Census Bureau. tl_2023_us_primaryroads. https://www.census.gov/cgi-bin/geo/shapefiles/index.php?year=2023&layergroup=Roads (2023).

[2] Muñoz Sabater, J. ERA5-Land monthly averaged data from 1950 to present. https://doi.org/10.24381/cds.68d2bb30 (2019).

[3] Baynes, J., Neale, A. & Hultgren, T. Improving intelligent dasymetric mapping population density estimates at 30m resolution for the conterminous United States by excluding uninhabited areas. *Earth Syst. Sci. Data* **14**, 2833-2849 (2022).

[4] OpenStreetMap contributors. Planet dump retrieved from https://planet.osm.org/. https://planet.osm.org (2024).

[5] Boeing, G. Modeling and analyzing urban networks and amenities with OSMnx. *Geogr. Anal.* **57**, 567-577(2025).

[6] Dewitz, J. and U.S. Geological Survey. National Land Cover Database (NLCD) 2019 Products (ver. 3.0, February 2024): U.S. Geological Survey data release. https://doi.org/10.5066/P9KZCM54 (2021).

[7] Dewitz, J. National Land Cover Database (NLCD) 2016 Products (ver. 3.0, November 2023):

U.S. Geological Survey data release. https://doi.org/10.5066/P96HHBIE (2019).

[8] QGIS Association. QGIS Geographic Information System. http://www.qgis.org (2025).

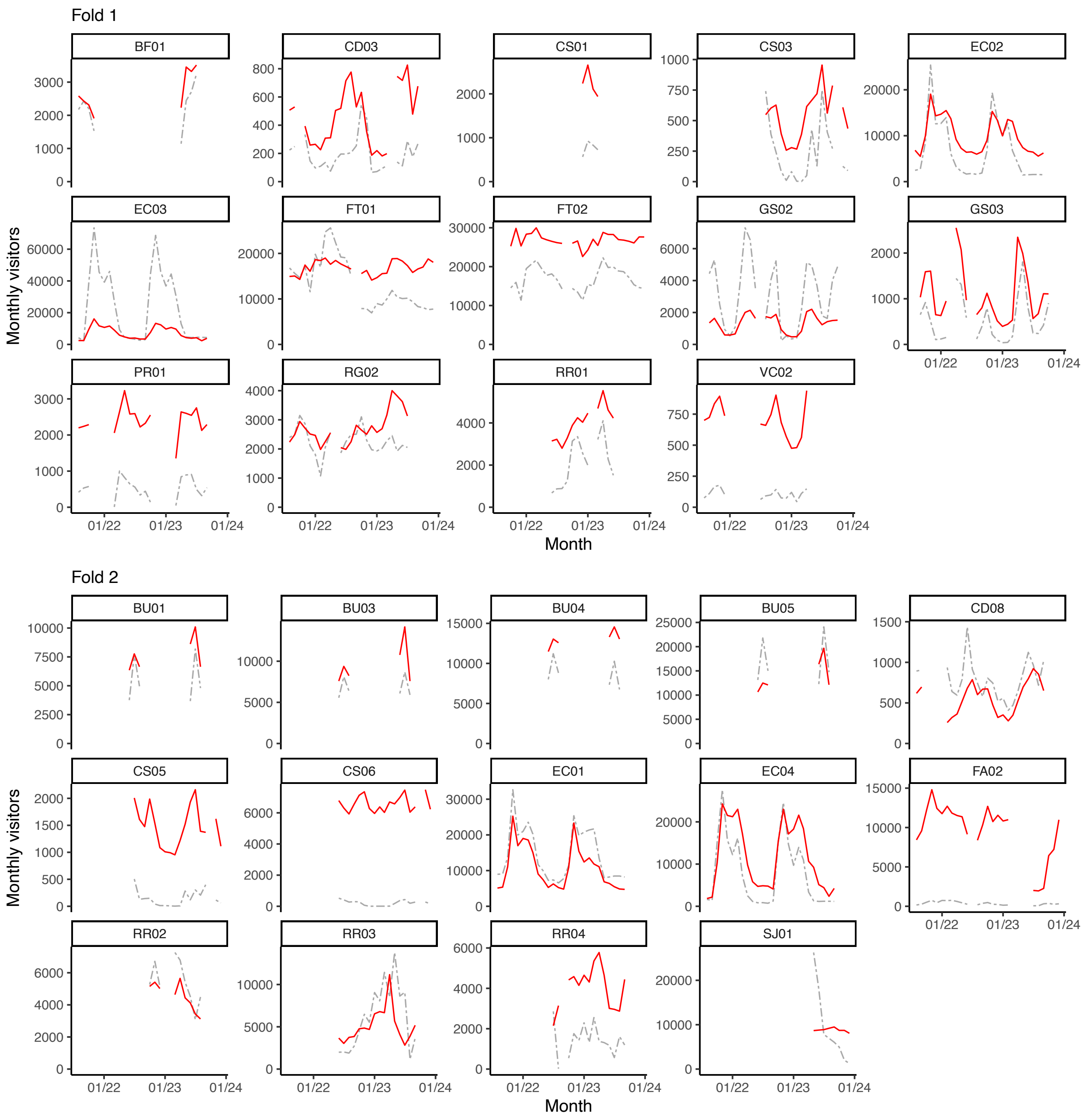

Supplementary Figure S1. Model 2 predictions of monthly visitors by site, grouped by the fold in which the site was held out as a test site. Grey dashed lines show observed values and solid red lines show predictions.

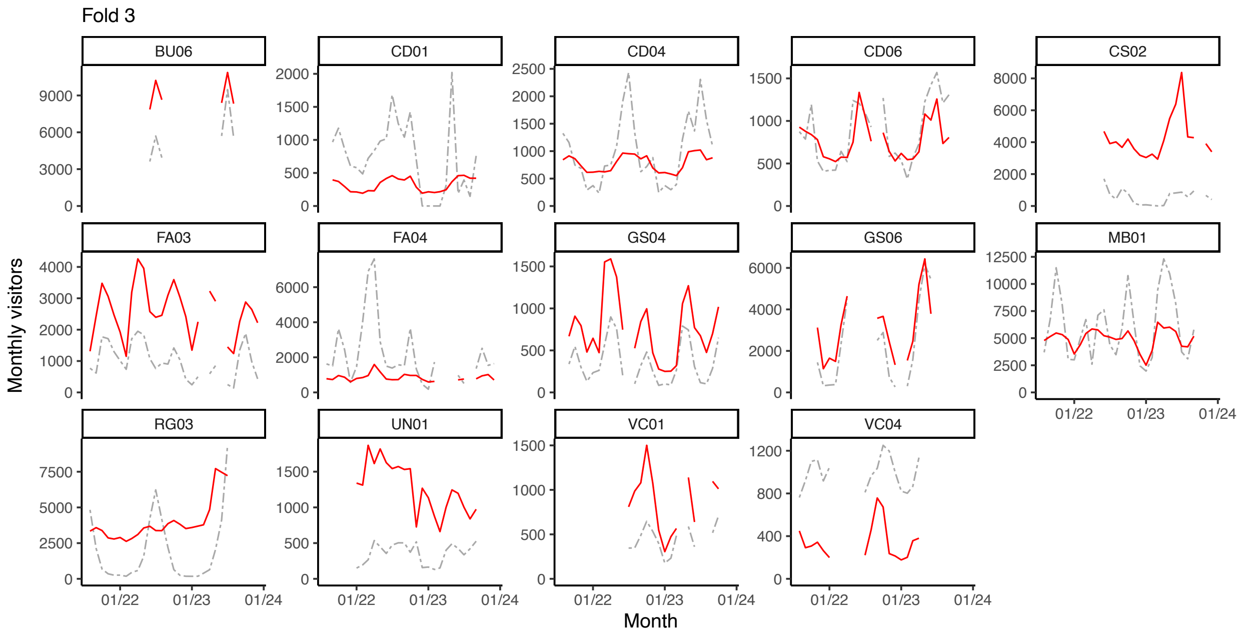

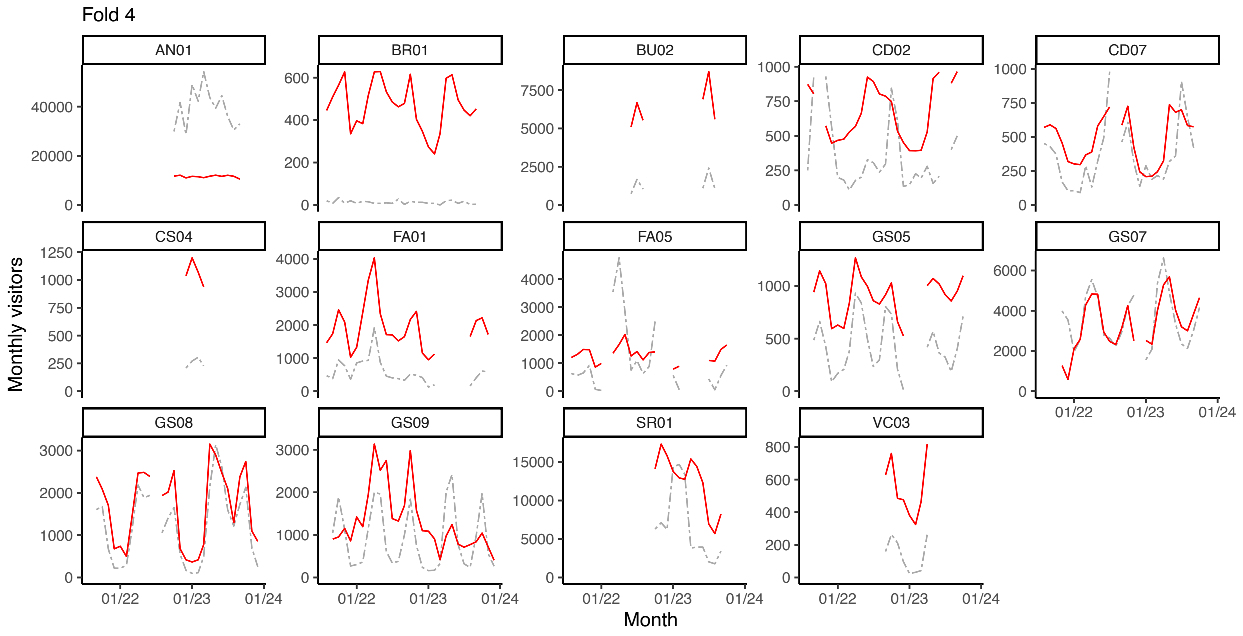

Supplementary Figure S1 continued. Model 2 predictions of monthly visitors by site, grouped by the fold in which the site was held out as a test site. Grey dashed lines show observed values and solid red lines show predictions.

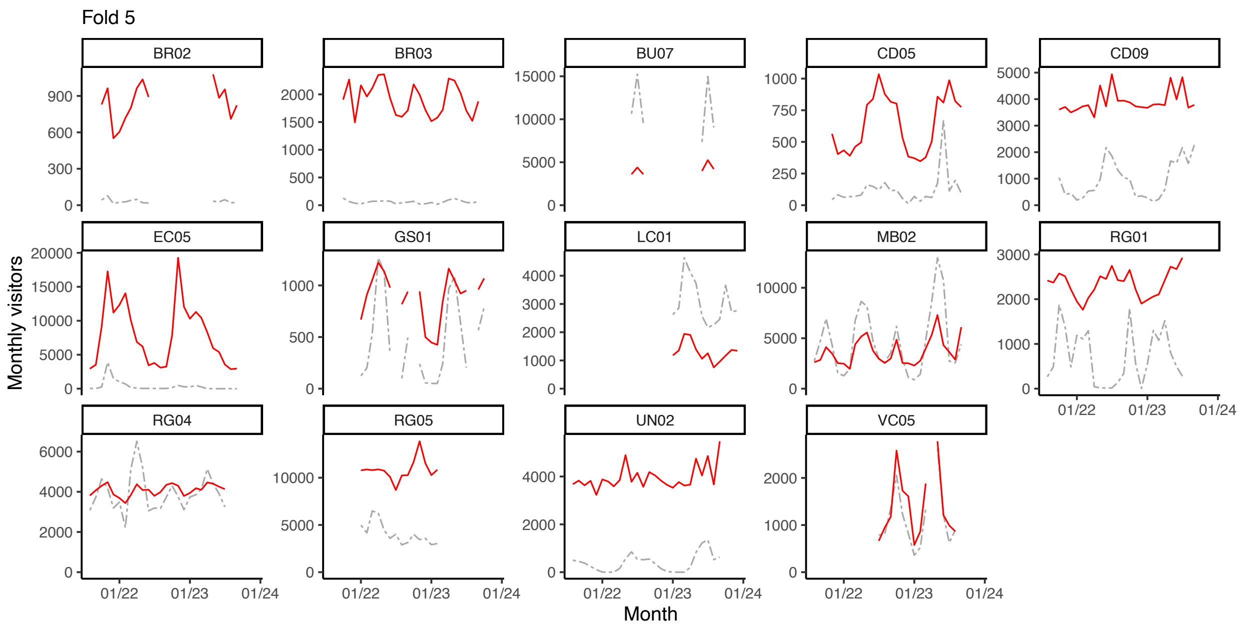

Supplementary Figure S1 continued. Model 2 predictions of monthly visitors by site, grouped by the fold in which the site was held out as a test site. Grey dashed lines show observed values and solid red lines show predictions.
